# Supplementary material for: Genomic inbreeding trends, influential sire lines and selection in the global Thoroughbred horse population
Source: Sci Rep. 2020 Jan 16;10:466. doi: 10.1038/s41598-019-57389-5 (PMC6965197; doi:10.1038/s41598-019-57389-5)
Supplement: Supplementary file 1 — Supplementary Text, Legends and Figures. [file 41598_2019_57389_MOESM1_ESM.pdf]

Genomic inbreeding trends, influential sire lines and selection in the global Thoroughbred horse population

Beatrice A. McGivney[1], Haige Han[1,2], Leanne R. Corduff[1], Lisa M Katz[3], Teruaki Tozaki[4], David E. MacHugh[2,5], Emmeline W. Hill[1,2,*]

[1]Plusvital Ltd, The Highline, Dun Laoghaire Business Park, Dublin, Ireland.

[2]UCD School of Agriculture and Food Science, University College Dublin, Ireland.

[3]UCD School of Veterinary Medicine, University College Dublin, Ireland.

[4]Genetic Analysis Department, Laboratory of Racing Chemistry, Utsunomiya, Tochigi, Japan.

[5]UCD Conway Institute of Biomolecular and Biomedical Research, University College Dublin, Ireland.

*Correspondence to Emmeline.Hill@ucd.ie

Stallion genotype reconstruction

Autosomal SNPs (46,478) were imputed for *n* = 127 sires based on genotyped progeny. Forty-three of these horses were also genotyped on at least one of the three SNP genotyping platforms. Concordance between inferred and SNP genotyping platform derived data ranged from 97.7-99.9% (S15 Table) with greater than 99% for 33 samples. Genetic distance was calculated using the pairwise identity by descent estimation function in Plink [1]. Distance is defined as (IBS2 +0.5*IBS1)/(n SNP pairs).

Inbreeding in the Thoroughbred population

Annual mean inbreeding levels are provided in S16-17 Tables and S12 Figure. Regional variation in inbreeding over time is shown in S13 Figure. Results of linear models of the relationship between inbreeding and year of birth within each region are provided in S14-S16 Figures. The average inbreeding value within the modern breeding population (horses born since 2010 with offspring) is F = 0.007 with no difference between stallions (*n* = 34) and mares (*n* = 75).

In addition to inferring inbreeding, analysis of runs of homozygosity (ROH) can identify genomic regions with the highest prevalence of ROH in the population potentially containing selected alleles. The top 1,000 SNPs ranked by the percentage of individuals with the SNP located within ROH are provided in S2-S3 Tables and S18 Figure. The top 1,000 SNPs were located within ROH in >53% of the individuals. Long ROH (>5 Mb), which reflect more recent inbreeding, on average account for 9.7% of the genome. This is consistent with a recent study by Fawcett el al. [2] which found that ROH segments >5 Mb account for an average of 233 Mb, or 10.4% of the genome.

However, ROH is not entirely informative on its own as alternate alleles of the same SNP may be identified in ROH in different animals. e.g. AA of the SNP BIEC2-5808 could be in a ROH in 20% of animals and GG in 26% of animals, appearing as ROH in 46% of animals. An additional limitation of ROH–based approaches lies in their underlying rule-based procedure. For instance the definition of the number of markers, segment length and proportion of allowed heterozygous markers is largely arbitrary

and also dependent on SNP density. Nonetheless, evaluation of the top SNPs ranked by the percentage of individuals homozygous for the SNP within ROH >1Mb identified the neurotrimin (*NTM*) gene region in >63% of horses (S2 Table). *NTM* has been highlighted as a hallmark of domestication in horses [3] and is associated with the chance of a Thoroughbred horse having a racecourse start [4], which may reflect its known function in neurodevelopment and the establishment of synapses [5].

Pedigree analysis

Ten generation pedigree data was ascertained for n = 7,262 Thoroughbred horses (www.pedigreequery.com). Within our dataset 97% of Thoroughbreds have *Northern Dancer* in their bloodline. This trend of rapid expansion of a dominant sire line is continuing. Within our dataset 410/743 horses born in ANZ between 2012 and 2017 can be traced back to *Danehill* (*Northern Dancer*'s grandson) within just three generations (i.e. 55% of Australian horses related through *Danehill* as a great-grandsire or closer). Within our dataset 1147/3298 horses born in EU between 2012 and 2017 can be traced back to *Sadler's Wells* (*Northern Dancer*'s son) within just three generations (i.e. 35% of EUR horses related through *Sadler's Wells* as a great-grandsire or closer). While pedigree data can be useful in highlighting broad trends in breeding practices multiple studies have shown that pedigree-based estimates of inbreeding and relatedness are less accurate than genomic methods [6-8]. This disparity between pedigree and genomic relatedness can be seen among *Danehill* descendants - only those with *Danehill* appearing once in their pedigree were included, n = 698 horses with year of birth (YOB) ranging from 1992 - 2017. A simplified pedigree-based estimation was used where relatedness to sire was estimated to be 0.5, relatedness to grandsire was estimated to be 0.25 and relatedness to great grandsire was estimated to be 0.125. Within the F1 generation (*Danehill* is sire; n = 18) genomic relatedness calculated using the IBD function in Plink [1] is close to 0.5, in agreement with pedigree-based estimates. However, in subsequent generations there are substantial deviations from the pedigree estimate. Within the F2 generation (*Danehill* is grandsire; n = 345) genomic relatedness ranged from 0.12 to 0.36 in contrast to pedigree-based estimates of 0.25, and 59% of horses from the F3 generation were as closely related to *Danehill* as those from the F2 generation (S19 Figure).

As a small number of sire lines are dominating Thoroughbred pedigrees there are also concerns regarding the loss of bloodlines with speculation that *The Byerley Turk* bloodline may soon be lost. Therefore it was of interest to look in detail at some of the remaining descendants of *The Byerely Turk* standing at stud today. We obtained blood samples from five stallions which trace back to *The Byerely Turk* on the male bloodline. These five stallions did not cluster separately on any of the PCA plots and inbreeding levels were comparable to those of other horses from the same regions and with the same year of birth. Recent analysis of Y chromosome variation [9] has established that there are likely more true *The Byerley Turk* lineages in the present population than previously thought; horses descended from *Galopin*, previously attributed to the *Darley Arabian* male lineage, in fact are descendants of *The Byerley Turk*.

CSS results

Genome-wide distribution of the smoothed CSS ($-\log_{10}P$) for the comparison of the Elite Thoroughbred (TBE) *versus* Thoroughbred founder populations identified 15 significant candidate selected genomic regions (S1 Table, S17 Figure), defined as clusters of ≥5 SNPs among the top 1% of the smoothed CSS statistic result ($-\log_{10}P$). 462 genes were identified underlying these selection peaks and flanking regions (± 0.5 Mb) and 387 were used as input for IPA analysis. The top canonical pathway identified was Airway Inflammation in Asthma. A full list of pathways is provided in S12 Table.

Effective population size

The pruned set of 9,212 SNPs was used for the calculation of effective population size (Ne). To estimate Ne, plink-formatted data was first converted to GENEPOP format using PGDSpider 2.1.1.5 [10]. Then the LD method in NeEstimator2x [11] was used to calculate Ne using the converted GENEPOP data as input. Ne was calculated for global thoroughbreds, individual region (ANZ, EUR,NAM and SAfr) and stallions. Year of birth for the horses used for Ne calculation were restricted to 2013 -2017.

Pops	Year of birth	N horses	Ne values	N SNPs used
ANZ	2013-2017	1041	197	9212
EUR	2013-2017	1293	198	9212
NAM	2013-2017	498	226	9212
SAfr	2013-2017	495	93	9212
Global	2013-2017	3341	330	9212

References

1 Chang, C. C. *et al.* Second-generation PLINK: rising to the challenge of larger and richer datasets. *Gigascience* **4**, 7, doi:10.1186/s13742-015-0047-8 (2015).

2 Fawcett, J. A. *et al.* Genome-wide SNP analysis of Japanese Thoroughbred racehorses. *PLoS One* **14**, e0218407, doi:10.1371/journal.pone.0218407 (2019).

3 Schubert, M. *et al.* Prehistoric genomes reveal the genetic foundation and cost of horse domestication. *Proceedings of the National Academy of Sciences* **111**, E5661-E5669, doi:10.1073/pnas.1416991111 (2014).

4 McGivney, B. A. *et al.* A genomic prediction model for racecourse starts in the Thoroughbred horse. *Anim Genet* **50**, 347-357, doi:10.1111/age.12798 (2019).

5 Mazitov, T., Bregin, A., Philips, M. A., Innos, J. & Vasar, E. Deficit in emotional learning in neurotrimin knockout mice. *BEHAVIOURAL BRAIN RESEARCH* **317**, 311-318, doi:10.1016/j.bbr.2016.09.064 (2017).

6 Kardos, M., Luikart, G. & Allendorf, F. W. Measuring individual inbreeding in the age of genomics: marker-based measures are better than pedigrees. *Heredity (Edinb)* **115**, 63-72, doi:10.1038/hdy.2015.17 (2015).

7 Wang, J. Pedigrees or markers: Which are better in estimating relatedness and inbreeding coefficient? *Theor Popul Biol* **107**, 4-13, doi:10.1016/j.tpb.2015.08.006 (2016).

8 VanRaden, P. M., Olson, K. M., Wiggans, G. R., Cole, J. B. & Tooker, M. E. Genomic inbreeding and relationships among Holsteins, Jerseys, and Brown Swiss. *Journal of Dairy Science* **94**, 5673-5682, doi:https://doi.org/10.3168/jds.2011-4500 (2011).

9 Felkel, S. *et al.* The horse Y chromosome as an informative marker for tracing sire lines. *Sci Rep* **9**, 6095, doi:10.1038/s41598-019-42640-w (2019).

10 Lischer, H. E. & Excoffier, L. PGDSpider: an automated data conversion tool for connecting population genetics and genomics programs. *Bioinformatics* **28**, 298-299, doi:10.1093/bioinformatics/btr642 (2012).

11 Do, C. *et al.* NeEstimator v2: re-implementation of software for the estimation of contemporary effective population size (Ne) from genetic data. *Mol Ecol Resour* **14**, 209-214, doi:10.1111/1755-0998.12157 (2014).

Supplementary Table and Figure Legends

S1 Table 31 stallions ROH at ECA1 region. Horses that were genotyped have been de-identified. Horses for which genotypes were imputed from progeny genotypes are identified.

S2 Table: Clusters identified with ≥5 SNPs among the top 1% based on the smoothed CSS statistic result (-log10P) for TB versus TB founder population comparison

S3 Table: The top 1000 SNPs ranked by the % individuals with SNP located within runs of homozygosity (ROH) of > 1mb

S4 Table: The top 1000 SNPs ranked by the % individuals with SNP located within runs of homozygosity (ROH) of > 5mb

S5 Table: Canonical pathways identified in the set of genes underlying selection peaks and flanking regions (± 0.5 Mb) for Thoroughbred (TB) versus TB founder population comparison using IPA analysis

S6 Table: 50 top canonical pathways identified in the set of genes underlying selection peaks and flanking regions (± 0.5 Mb) for Thoroughbred (TB) versus TB founder population comparison using IPA analysis by manual curation

S7 Table: Summary of distribution of horses based on region of birth and year of birth

S8 Table: List of imputed sires and number of progeny used for imputation, Dist is the genetic distance calculated by the pairwise identity by descent defined as (IBS2 +0.5*IBS1)/(n SNP pairs).

S9 Table: The annual mean, SE, SD and CI were calculated based on the horse's year of birth for Inbreeding coefficient *F*.

S10 Table: The annual mean, SE, SD and CI were calculated based on the horse's year of birth for *ROHs*

S1 Figure: Thoroughbreds and breeds of origin PC1 v PC2

S2 Figure: Thoroughbreds and breeds of origin PC2 v PC3

S3 Figure: Global genetic variation in Thoroughbreds PC1 v PC2

S4 Figure: Global genetic variation in Thoroughbreds PC2 v PC3

S5 Figure: Stallions PC2 v PC3

S6 Figure: Within region genetic variation in Thoroughbreds – EUR PC1 v PC2

S7 Figure: Within region genetic variation in Thoroughbreds – EUR PC2 v PC3

S8 Figure: Within region genetic variation in Thoroughbreds – ANZ PC1 v PC2

S9 Figure: Within region genetic variation in Thoroughbreds – ANZ PC2 v PC3

S10 Figure: Within region genetic variation in Thoroughbreds – NAM PC1 v PC2

S11 Figure: Within region genetic variation in Thoroughbreds – NAM PC2 v PC3

S12 Figure: Global genetic variation of mean F_{IS} and F_{ROH} over time with standard error indicated by error bars.

S13 Figure: Regional genetic variation in annual mean F_{IS} and F_{ROH}

S14 Figure: Linear regression fit for inbreeding by Year of birth in Australasia (ANZ)

S15 Figure: Linear regression fit for inbreeding by Year of birth in Europe (EUR)

S16 Figure: Linear regression fit for inbreeding by Year of birth in North America (NAM)

S17 Figure: Manhattan plot for CSS and smoothed CSS values, showing significant gene regions under selection for Thoroughbreds

S18 Figure: Manhattan plot of the distribution of runs of homozygosity (Greater than 1Mb in length and SNP located within ROH in > 20% of the population) in the Thoroughbred population.

S19 Figure: Comparison of pedigree-based estimates (x-axis) and the genomic estimate of relatedness (y-axis) of direct descendants of *Danehill*

S1 Figure
Thoroughbreds and breeds of origin PC1vPC2

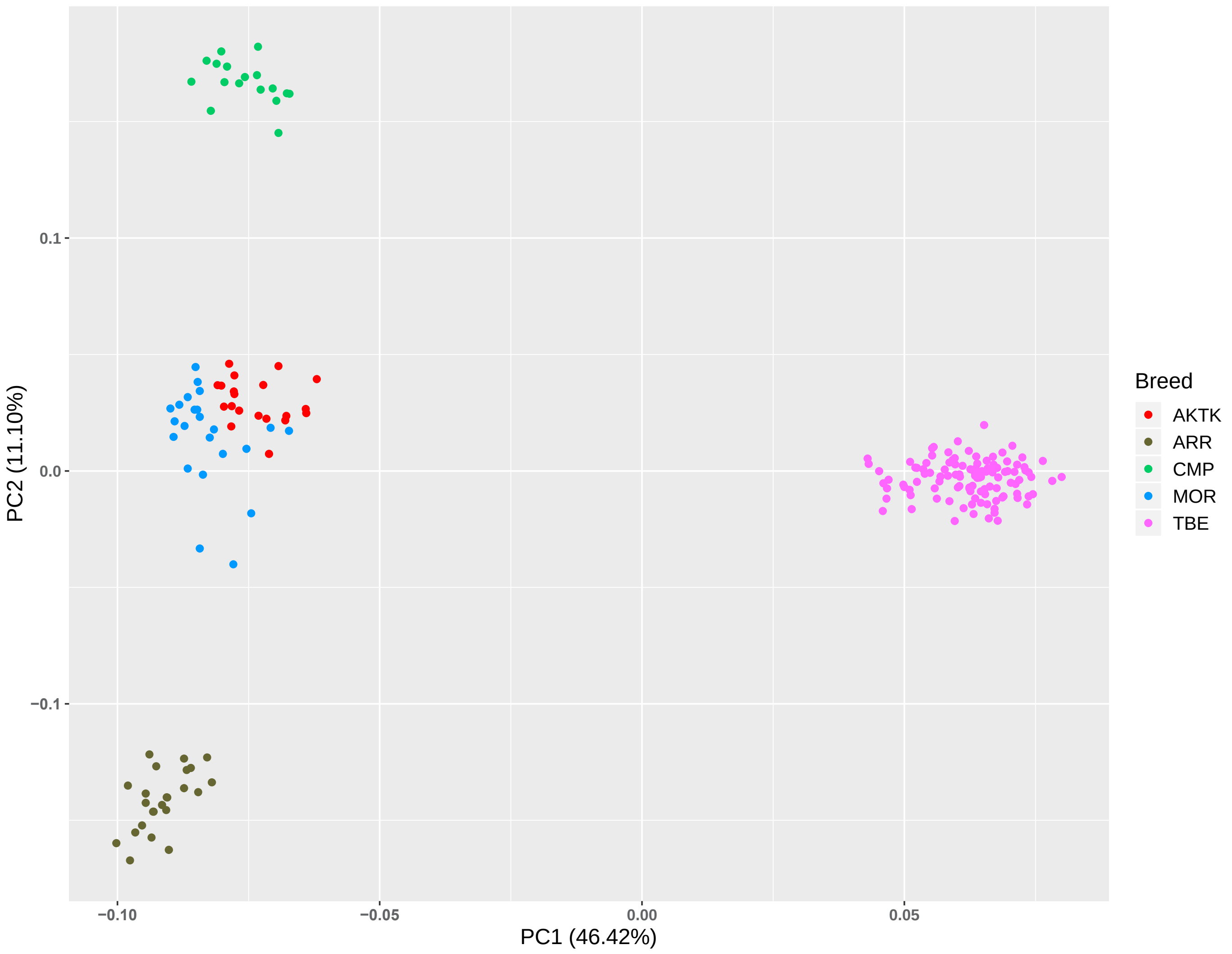

S2 Figure Thoroughbreds and breeds of origin PC2vPC3

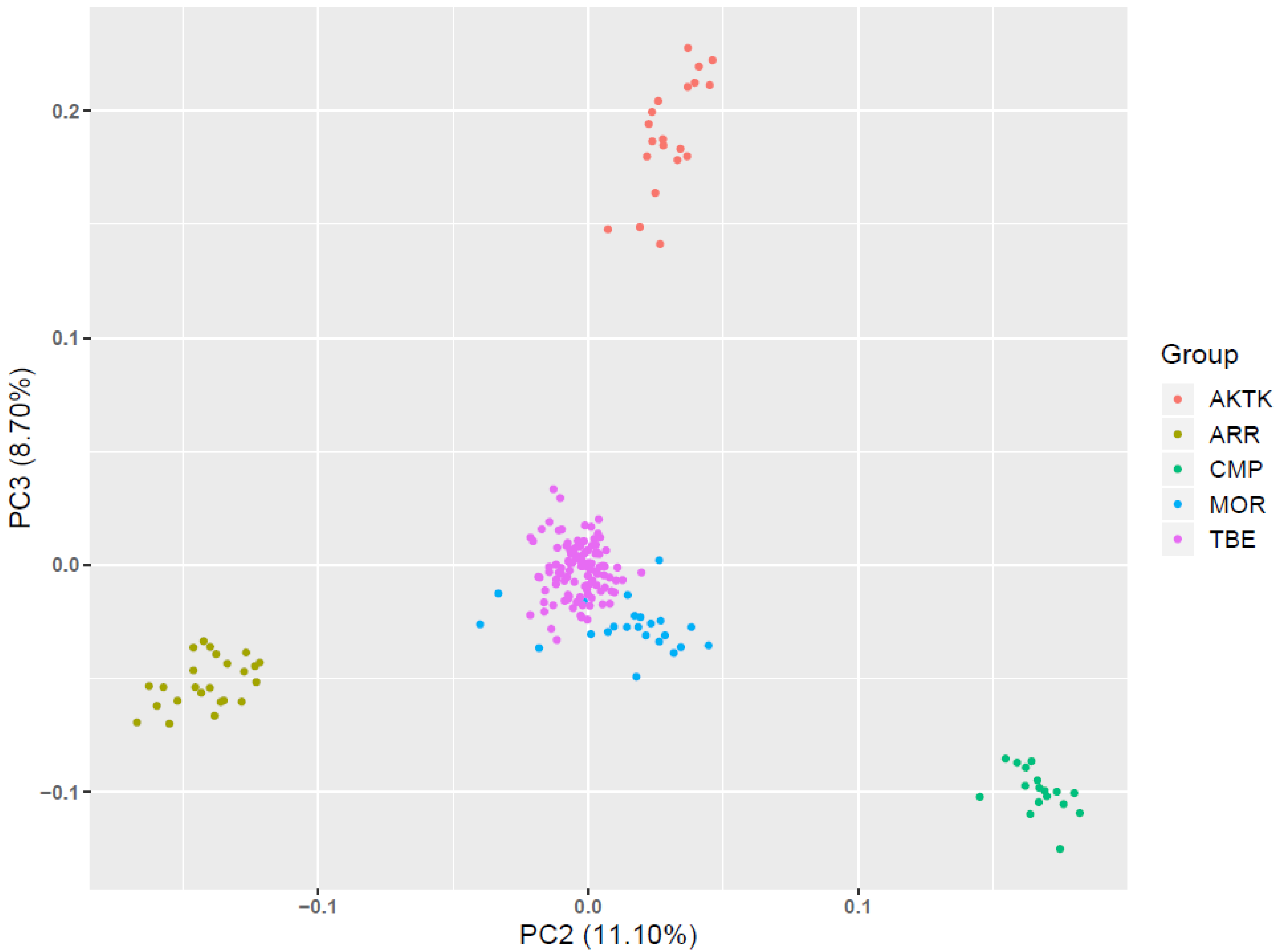

S3 Figure
Global variation in Thoroughbreds PC1 v PC2

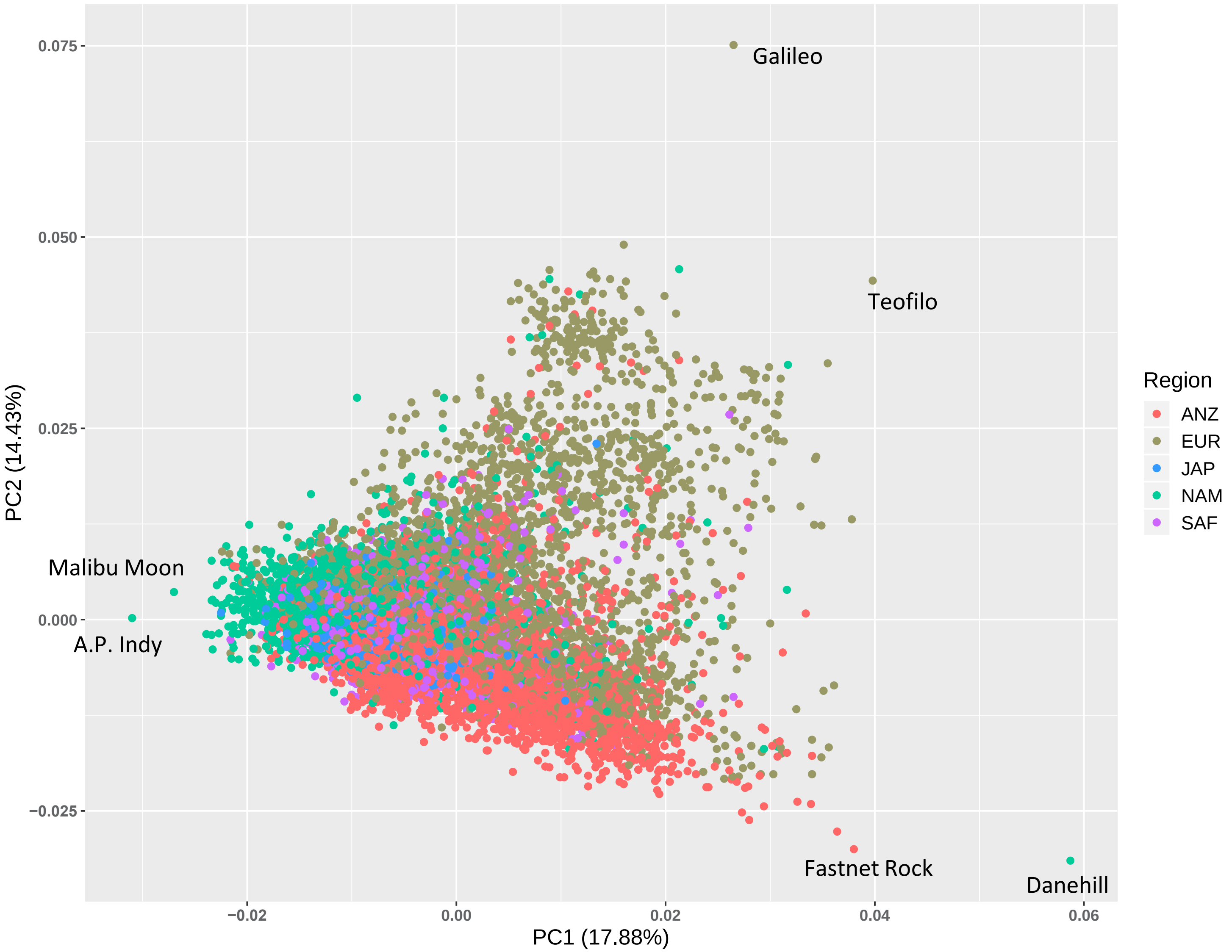

S4 Figure
Global variation in Thoroughbreds PC2 v PC3

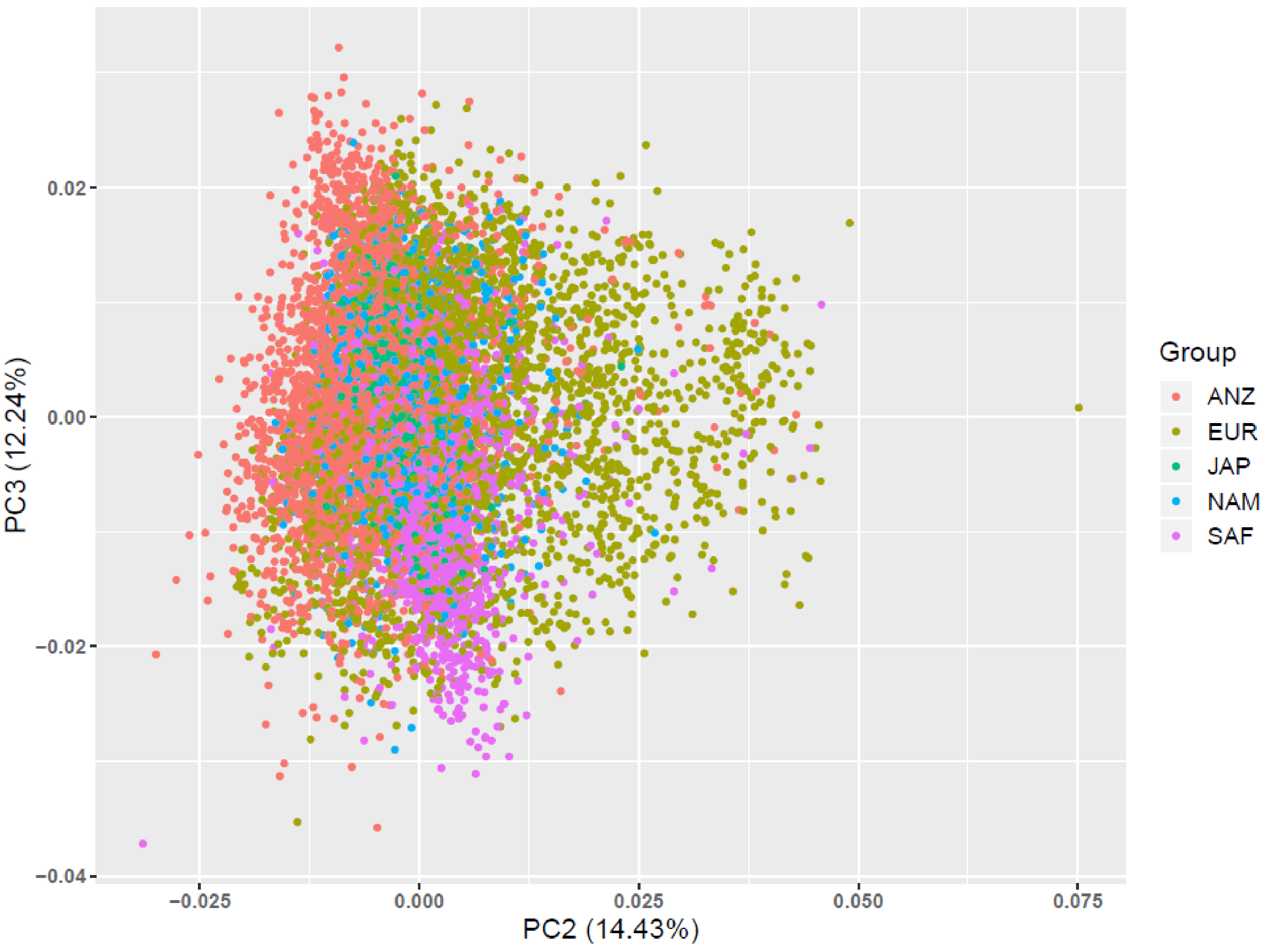

S5 Figure

Stallions (n = 305) PC2vPC3

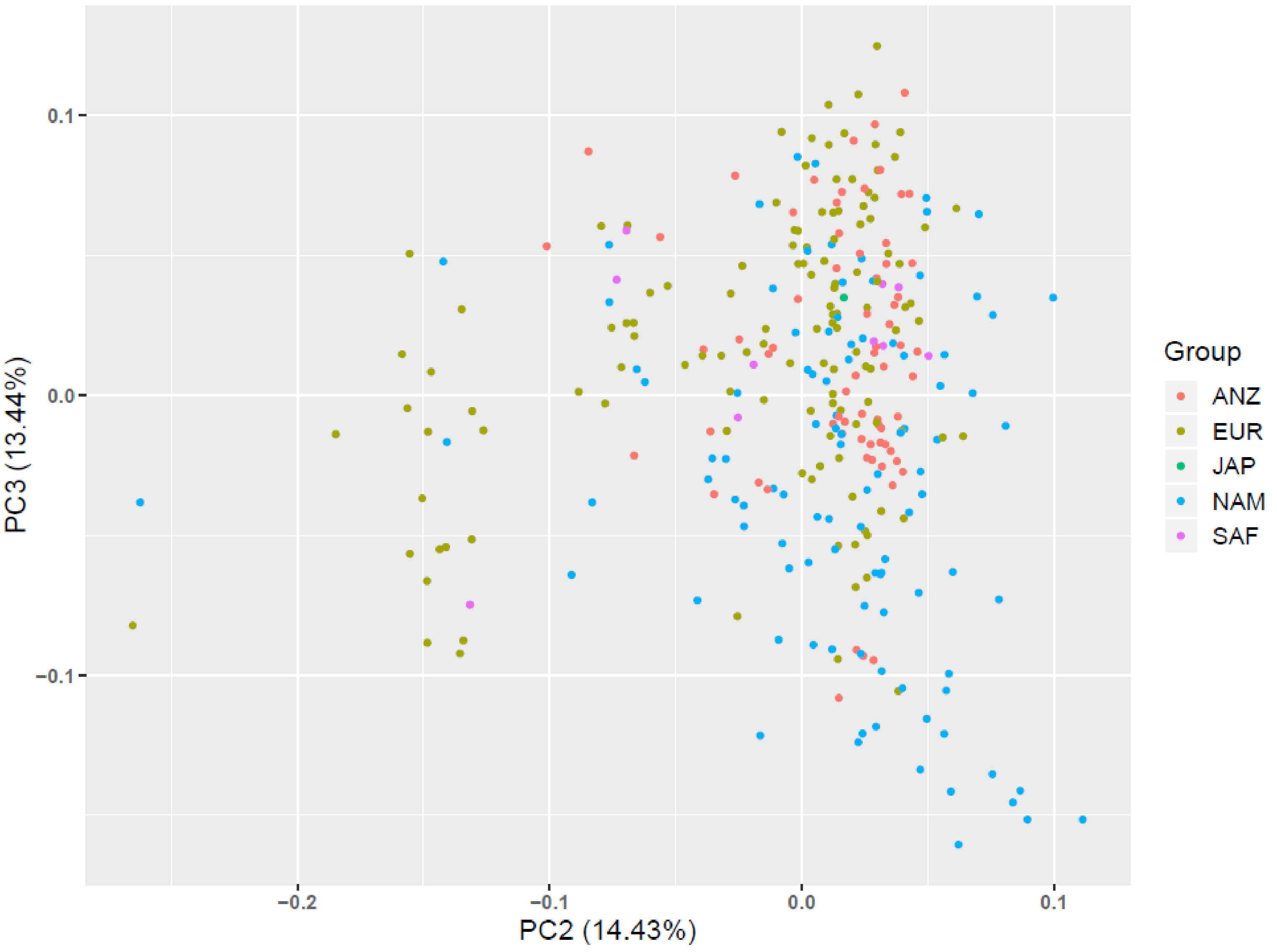

S6 Figure
Within region variation in Thoroughbreds – EUR
PC1vPC2

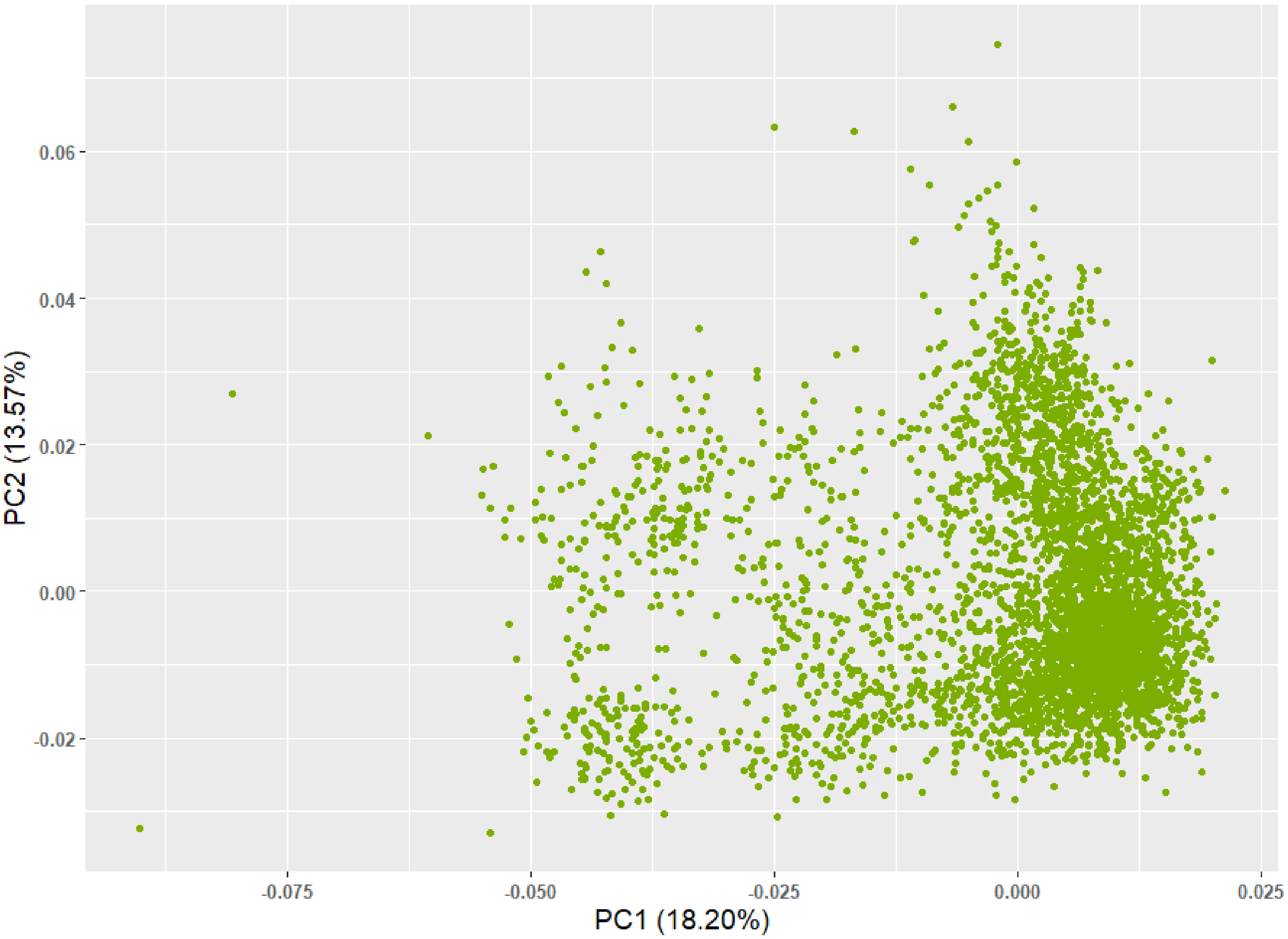

S7 Figure
Within region variation in Thoroughbreds – EUR
PC2vPC3

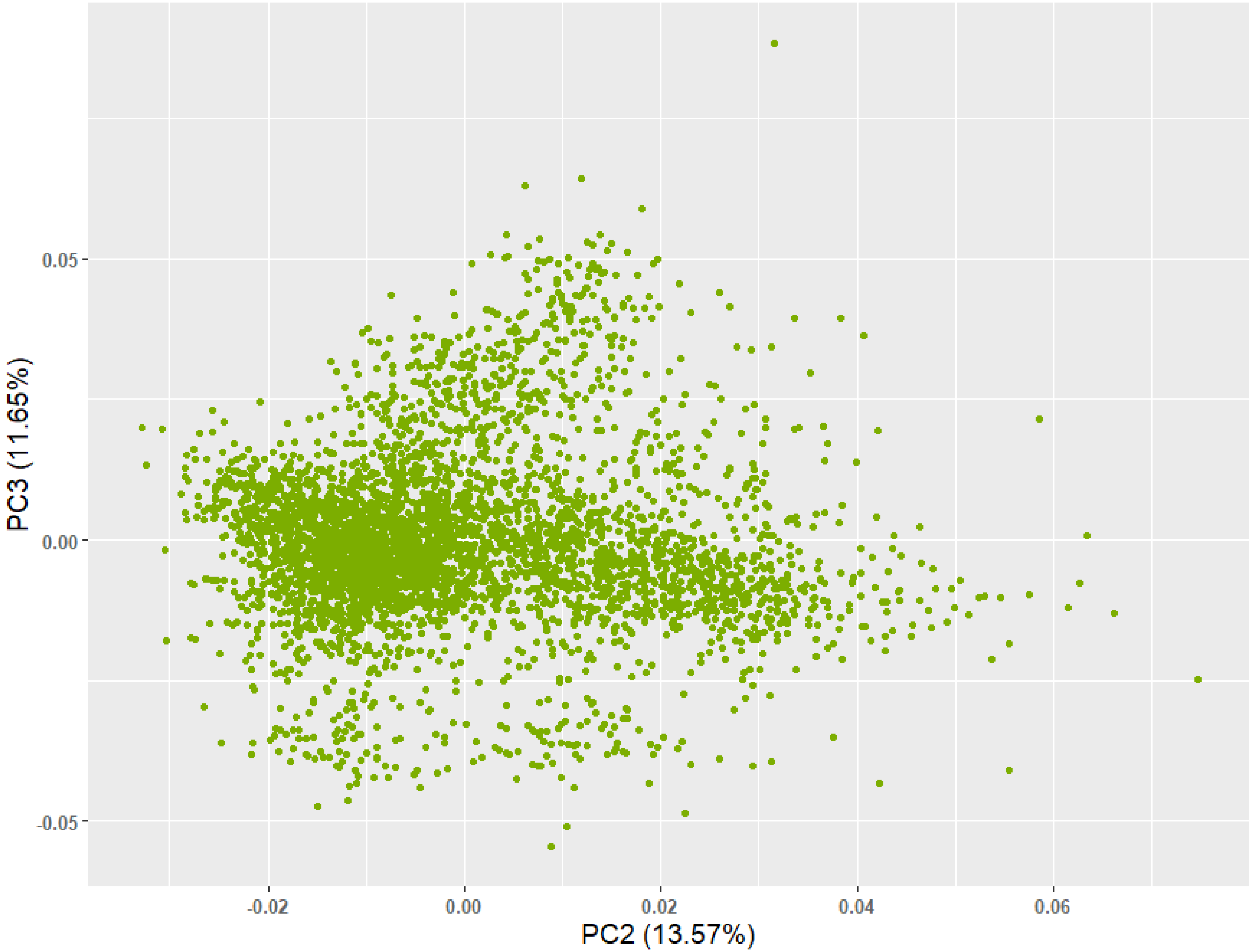

S8 Figure
Within region variation in Thoroughbreds – ANZ
PC1vPC2

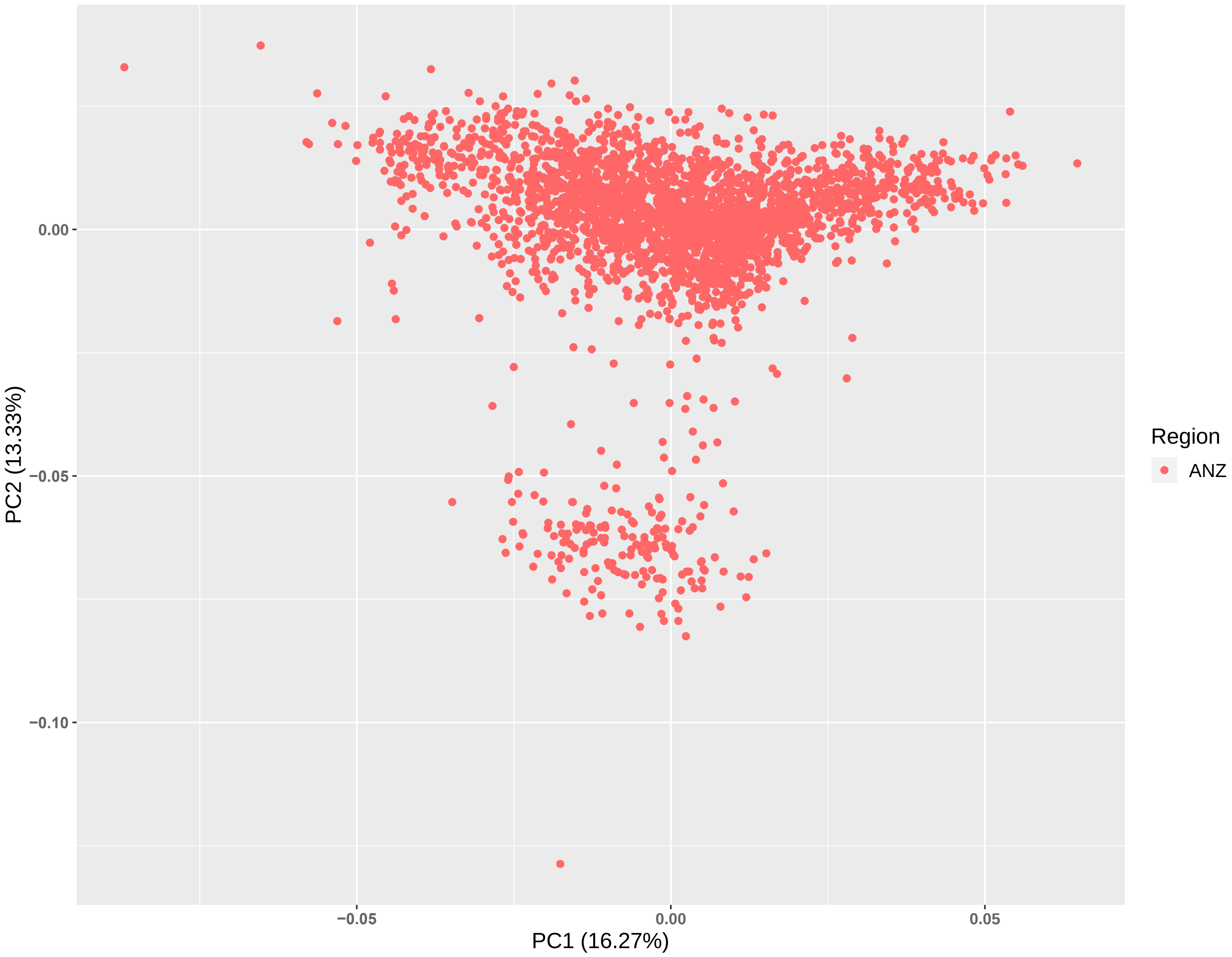

S9 Figure
Within region variation in Thoroughbreds – ANZ
PC2vPC3

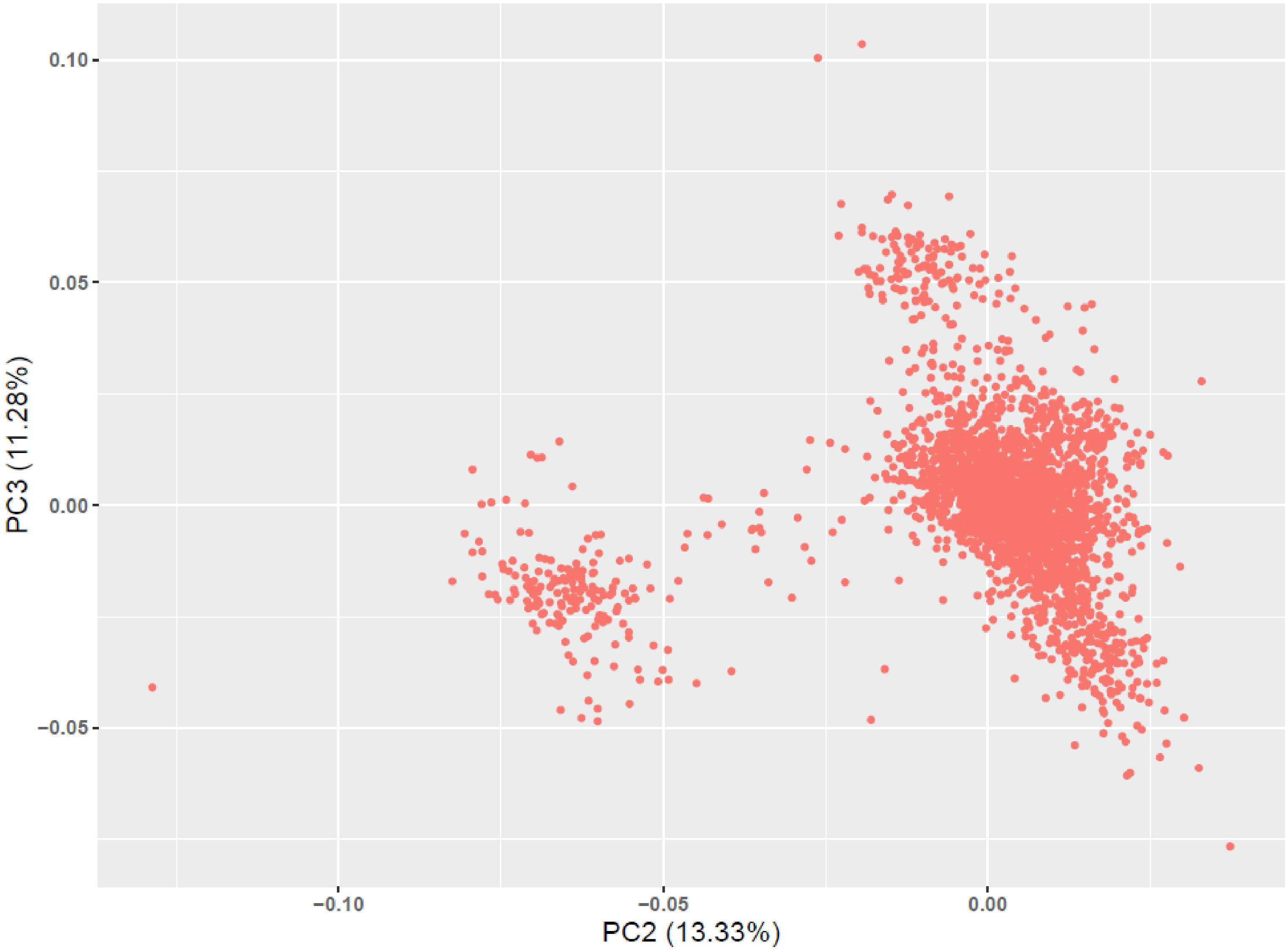

S10 Figure
Within region variation in Thoroughbreds – NAM
PC1vPC2

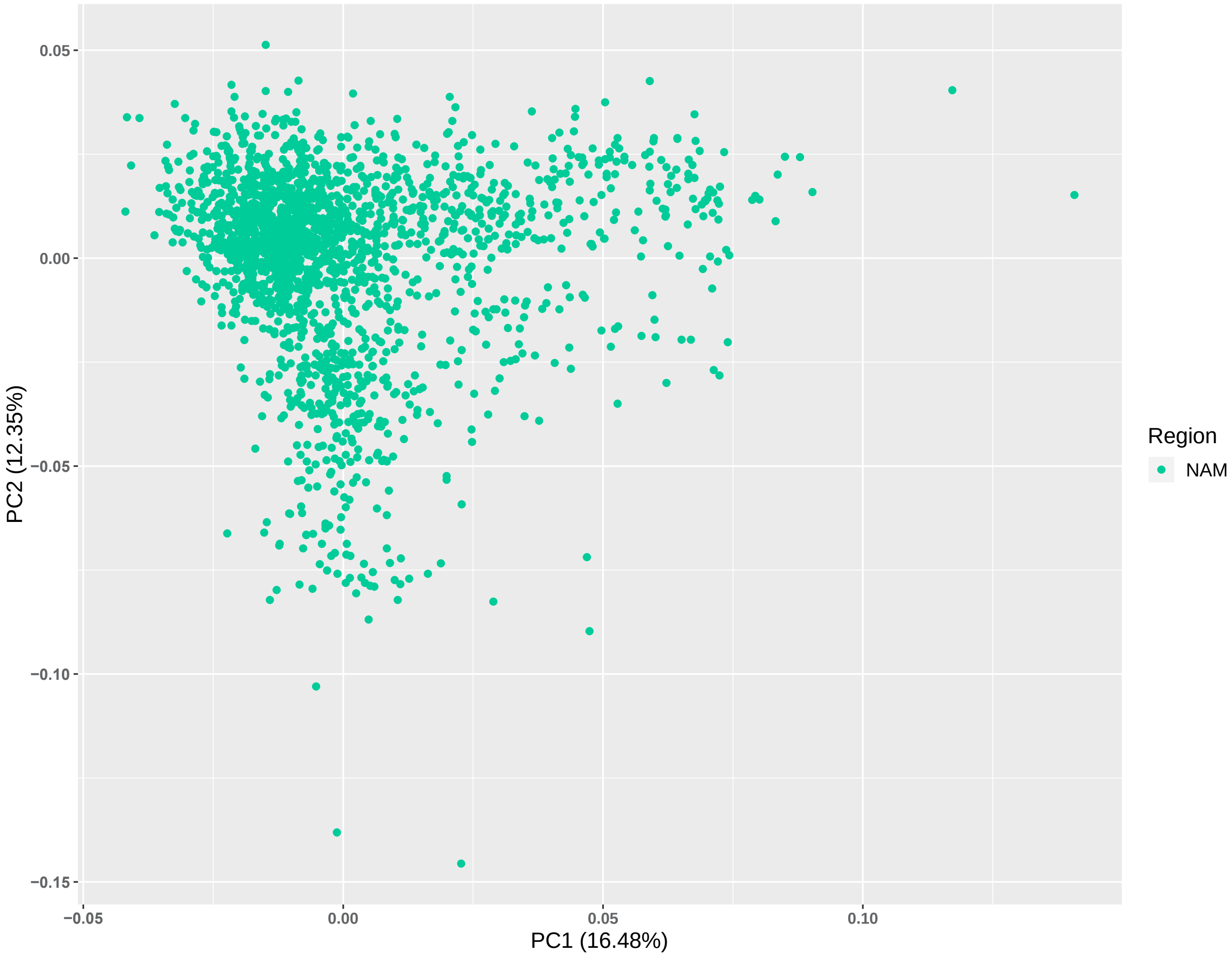

S11 Figure
Within region variation in Thoroughbreds – NAM
PC2vPC3

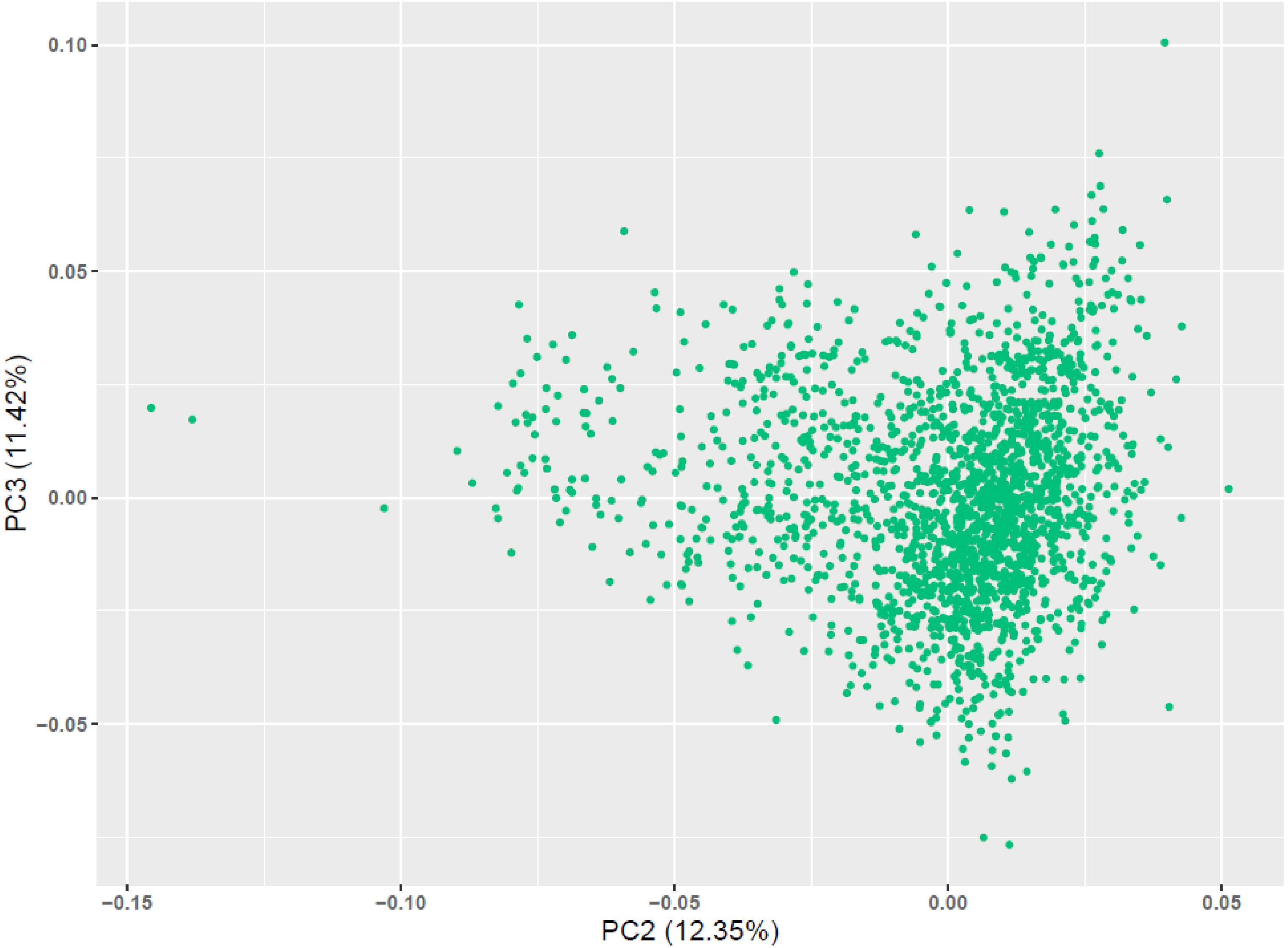

S12 Figure
Global variation in inbreeding over time

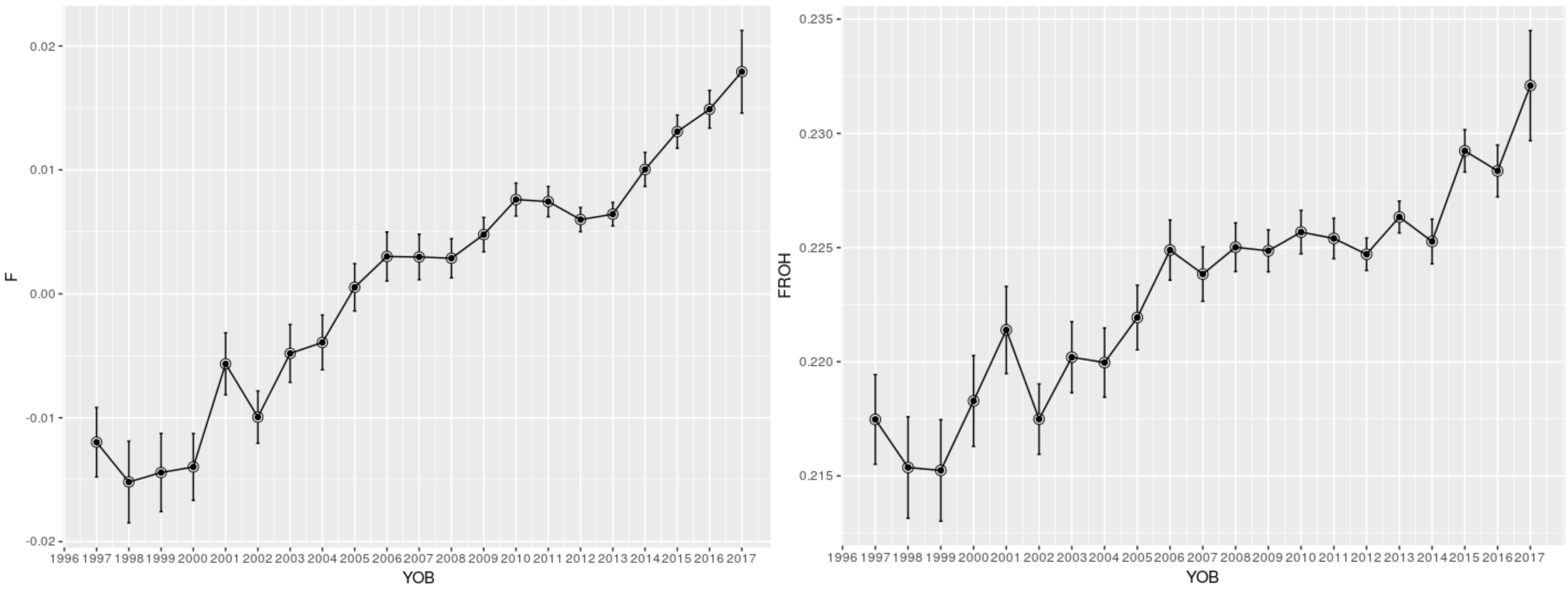

S13 Figure
Regional variation in inbreeding over time

Green – EUR
Blue – NAM
Red - ANZ

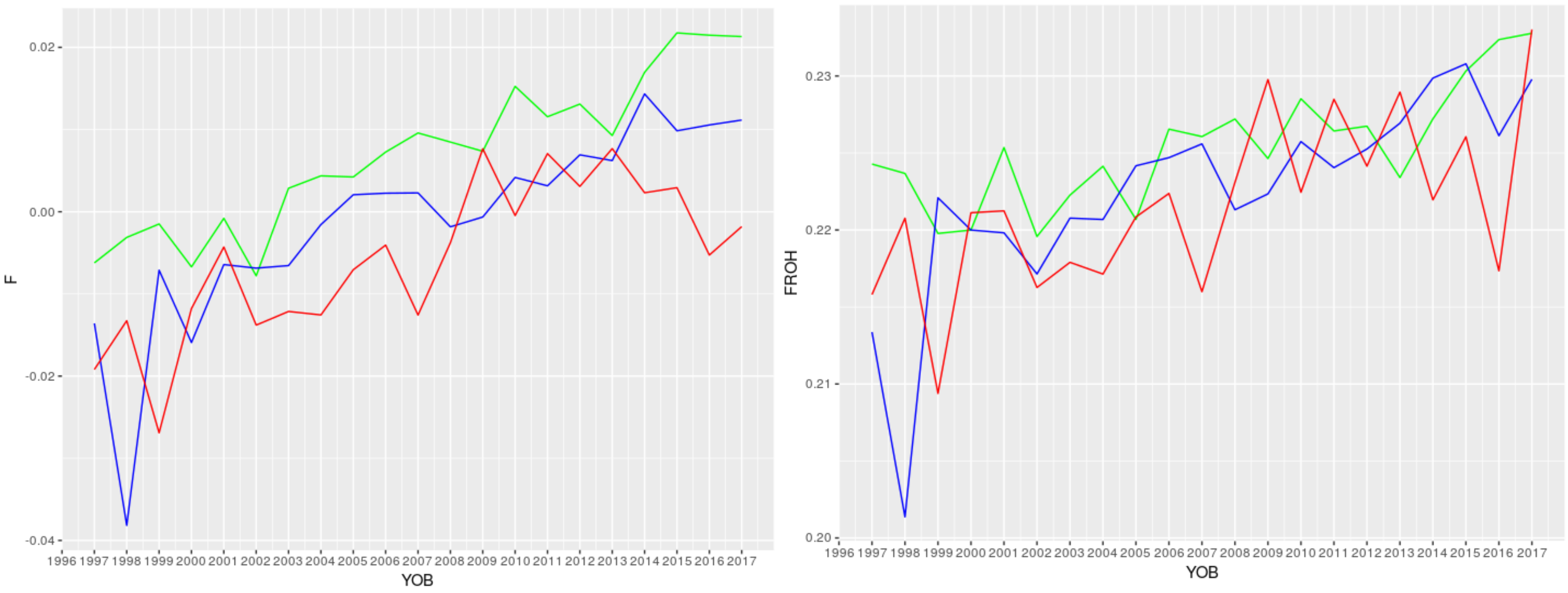

S14 Figure
Linear Regression fit for inbreeding by year of birth in Australasia (ANZ)

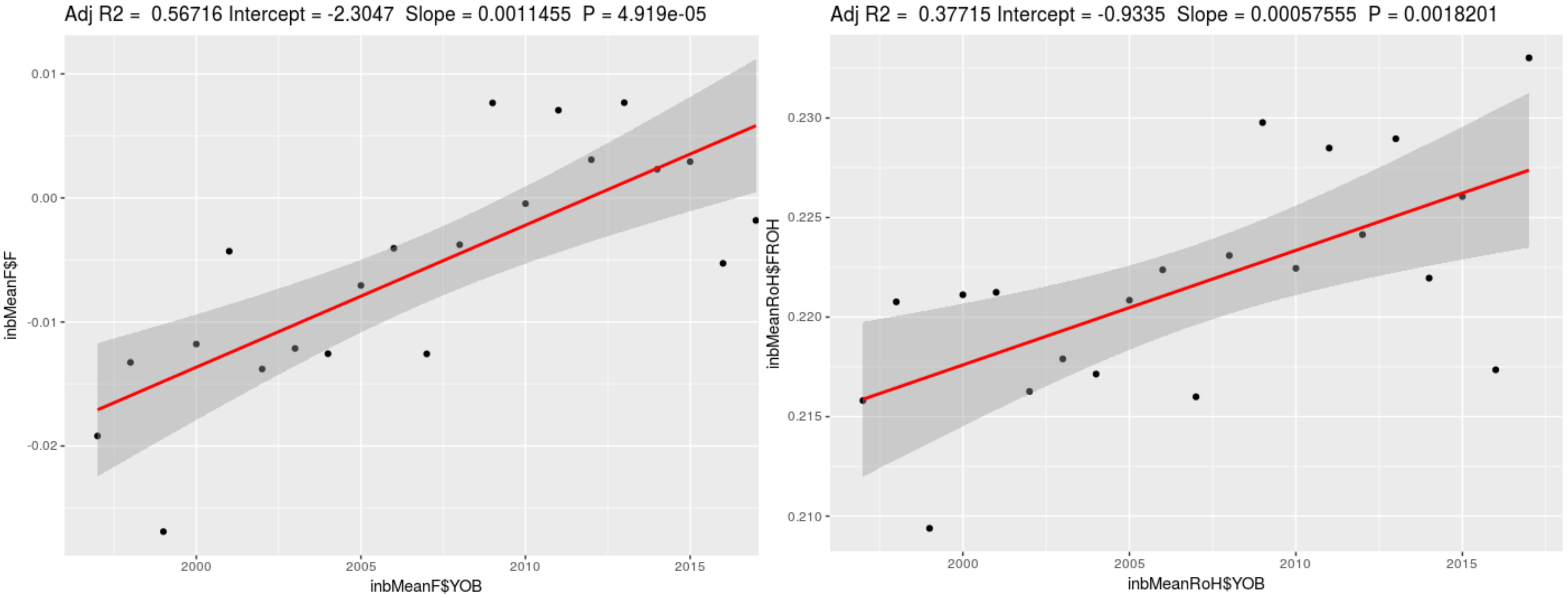

S15 Figure
Linear Regression fit for inbreeding by Year of birth in Europe (EUR)

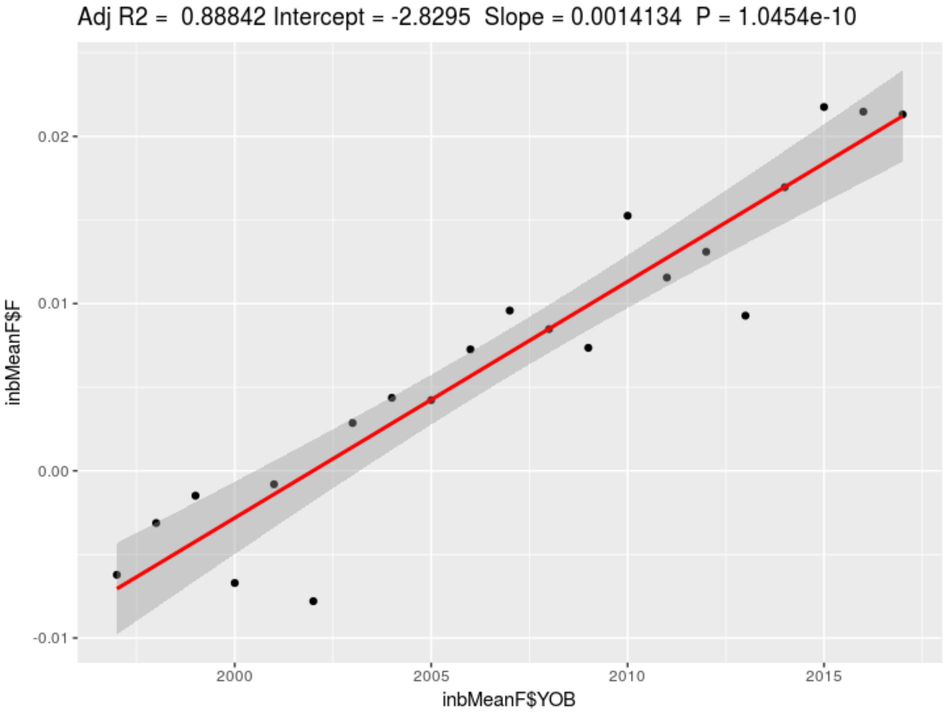

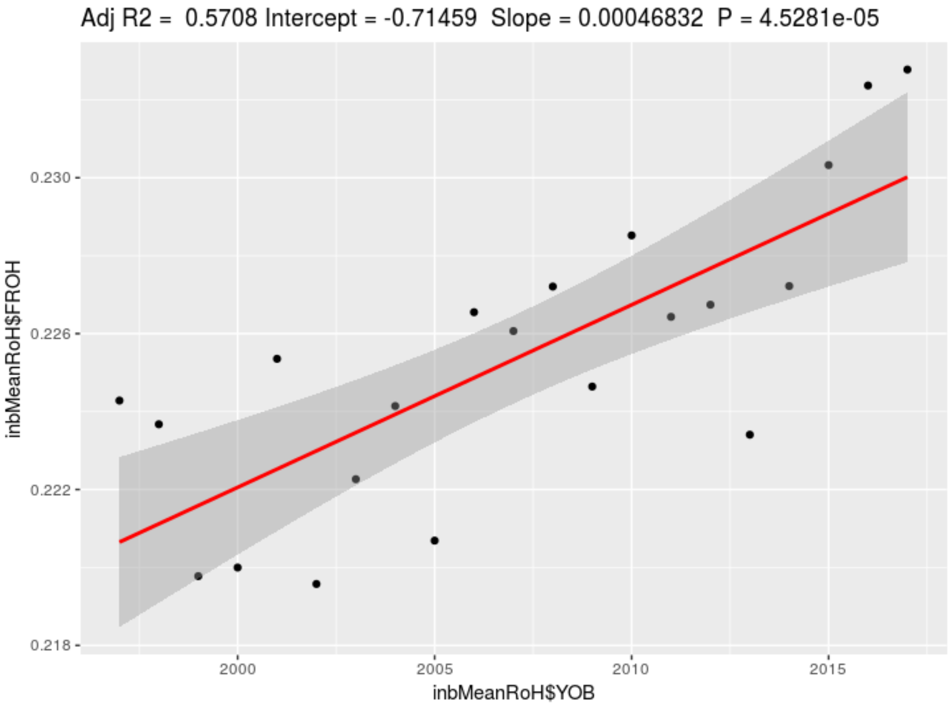

S16 Figure

Linear Regression fit for inbreeding by Year of birth in North America (NAM)

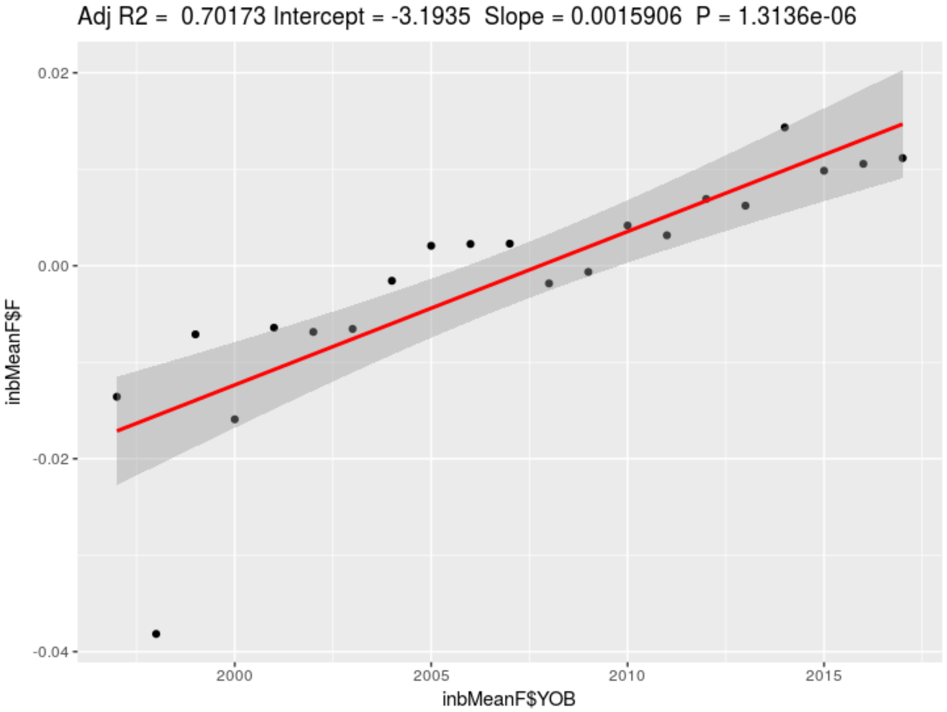

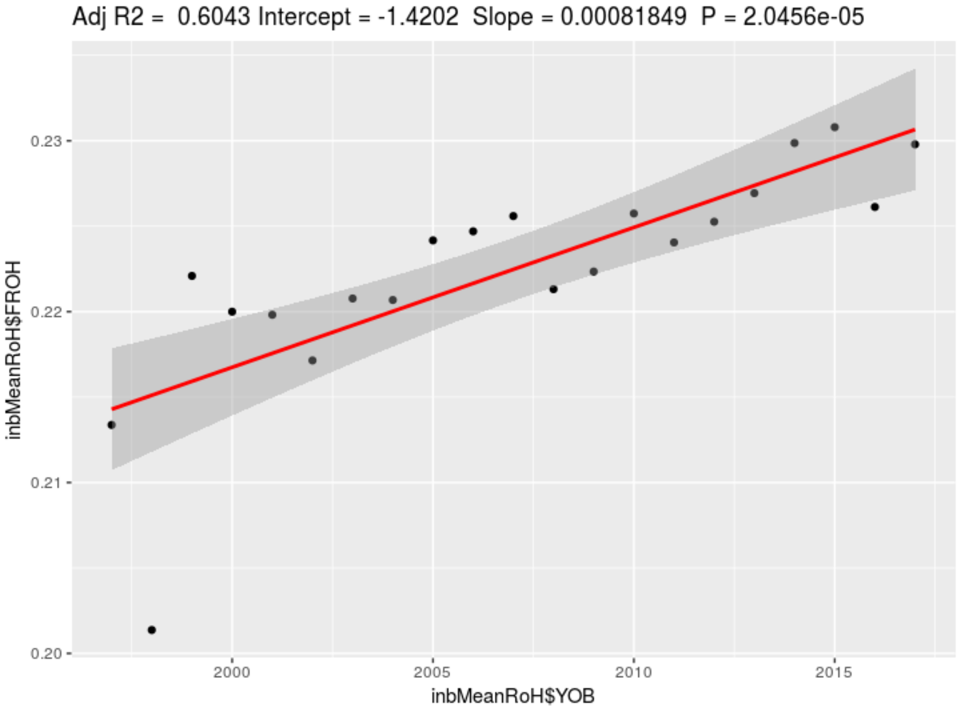

S17 Figure
Manhattan plot for CSS and smoothed CSS values, showing significant gene regions under selection for Thoroughbreds

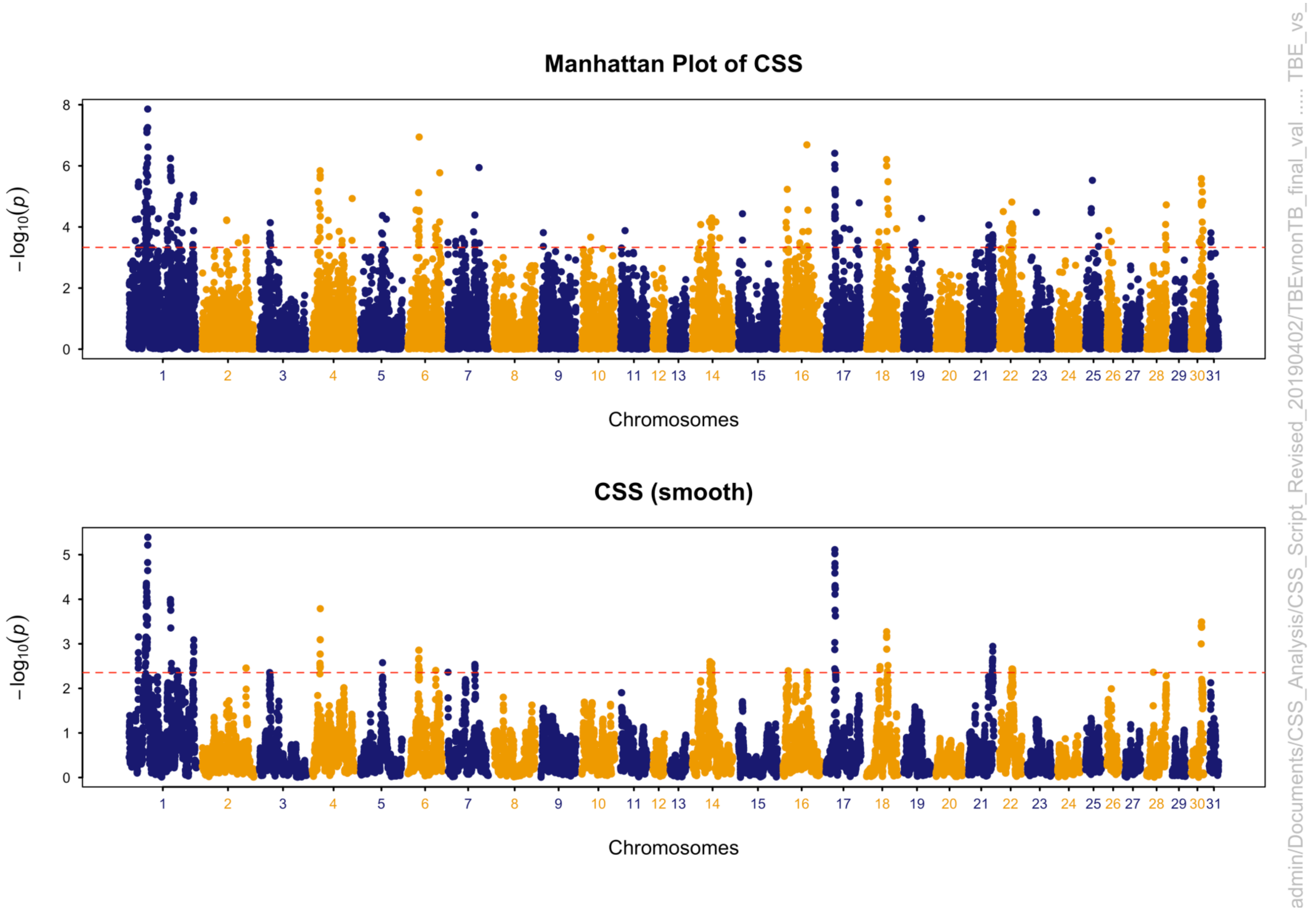

S18 Figure

Manhattan plot of the distribution of runs of homozygosity (Greater than 1mb in length and SNP located within ROH in > 20% of the population) in the Thoroughbred population.

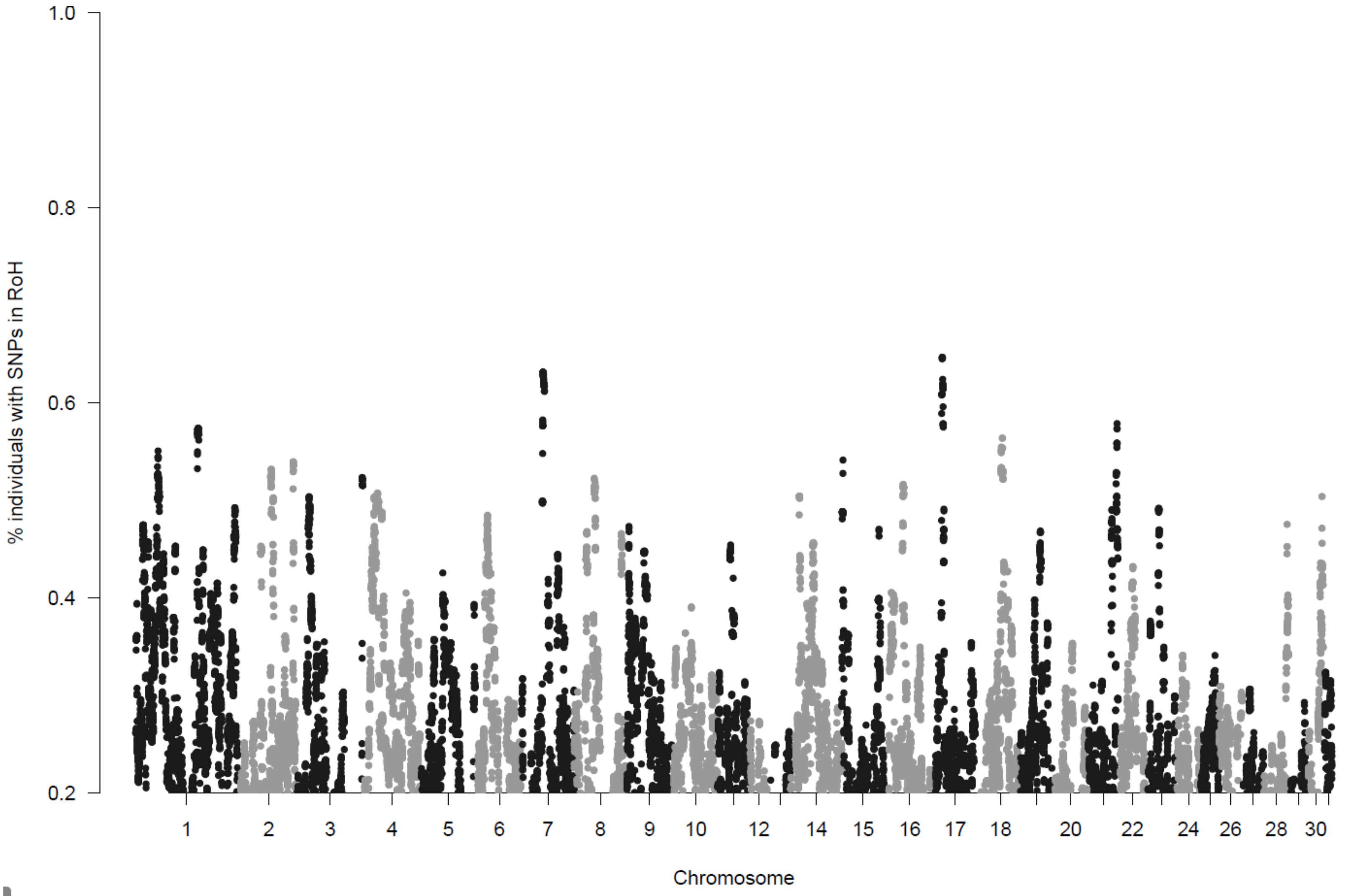

S19 Figure
Comparison of pedigree-based estimates (x-axis) and the genomic estimate of relatedness (y-axis) of direct descendants of *Danehill*

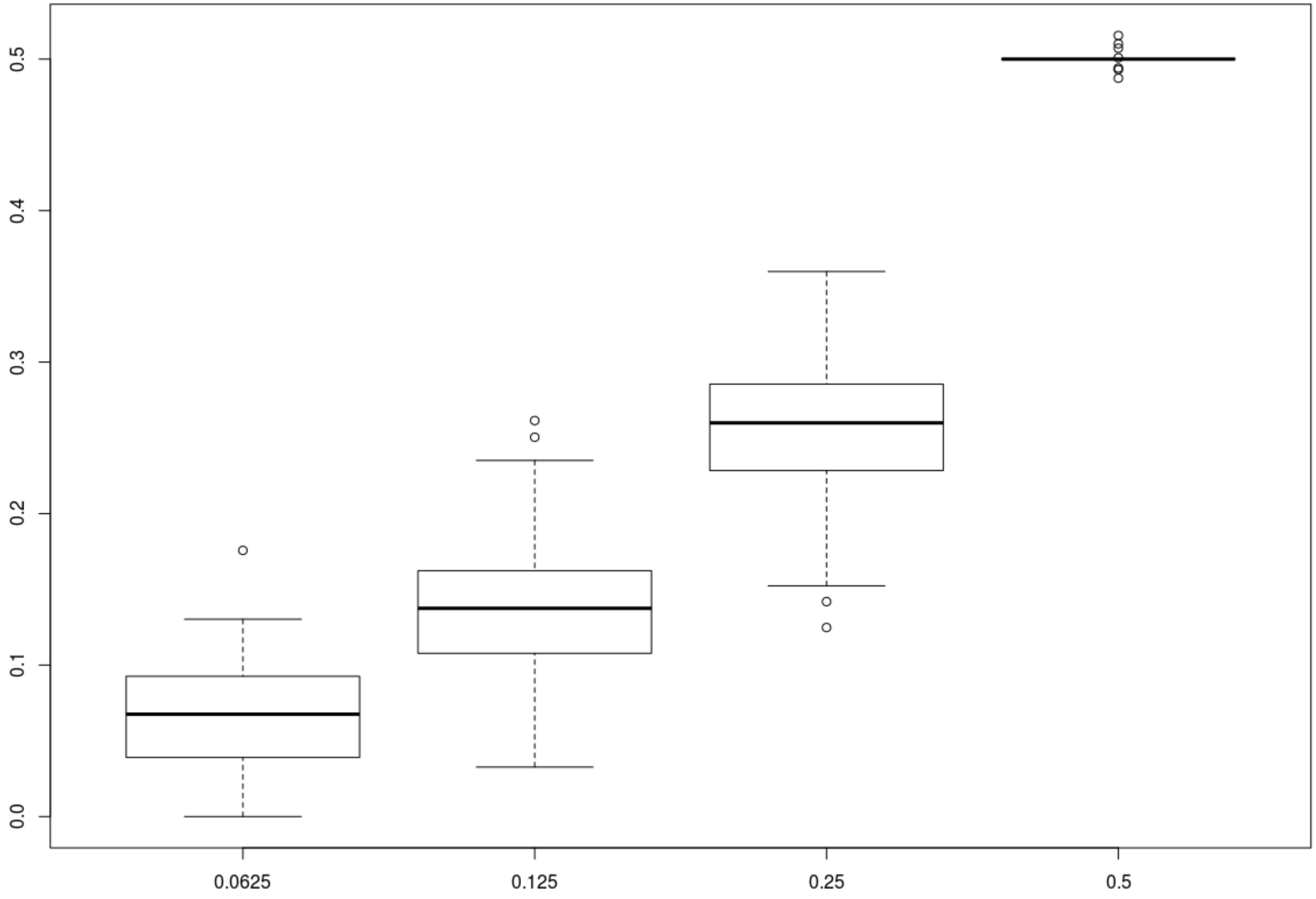

Within our dataset 410/743 horses born in AusNZ between 2012 and 2017 can be traced back to *Danehill* (*Northern Dancer* grandson) within just three generations i.e. 55% of Australian horses are related through *Danehill* as grandsire or closer.
